# Neuroanatomical differences in the memory systems of intellectual giftedness and typical development

**DOI:** 10.1002/brb3.2348

**Published:** 2021-10-14

**Authors:** Taylor Kuhn, Robin Blades, Lev Gottlieb, Kendra Knudsen, Christopher Ashdown, Laurel Martin‐Harris, Dara Ghahremani, Bianca H. Dang, Robert M. Bilder, Susan Y. Bookheimer

**Affiliations:** ^1^ Department ofPsychiatry and Biobehavioral Sciences UCLA 635 Charles E Young Dr, South Los Angeles CA 90025 USA

**Keywords:** connectivity, intellectual giftedness, learning strategies, memory systems, neuroanatomy

## Abstract

**Introduction:**

Studying neuro‐structural markers of intellectual giftedness (IG) will inform scientific understanding of the processes helping children excel academically.

**Methods:**

Structural and diffusion‐weighted MRI was used to compare regional brain shape and connectivity of 12 children with average to high average IQ and 18 IG children, defined as having IQ greater than 145.

**Results:**

IG had larger subcortical structures and more robust white matter microstructural organization between those structures in regions associated with explicit memory. TD had more connected, larger subcortical structures in regions associated with implicit memory.

**Conclusions:**

It was found that the memory systems within brains of children with exceptional intellectual abilities are differently sized and connected compared to the brains of typically developing children. These different neurodevelopmental trajectories suggest different learning strategies. A spectrum of intelligence types is envisioned, facilitated by different ratios of implicit and explicit system, which was validated using a large external dataset.

## Graphical abstract

A double dissociation was found between the size and connectomics of explicit and implicit memory systems within brains of children with exceptional intellectual abilities and the brains of typically developing children. These divergent neurodevelopmental trajectories appear to support different learning systems and therefore strategies. A heuristic for the spectrum of intelligence types was envisioned, facilitated by different ratios of neural implicit and explicit memory systems, validated using a large external dataset.

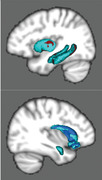

## 1 INTRODUCTION

Despite their exceptional learning and testing aptitude, we know surprisingly little about how the learning and memory systems in intellectually “gifted” brains develop and function. Children with exceptional intellect typically have more efficient memory functions, larger and more intricately organized knowledge bases and are capable of using more complex cognitive strategies which rely on these memory and semantic structures to solve problems more quickly or at an earlier age than typically developing (TD) children (Ali et al., 2003, Athanasakis et al., 2014, Colom et al., 2004, Davidson, 1986). Thus, they are able to generalize knowledge across domains, make intuitive leaps (Desco et al., 2011), and spontaneously use selective learning as well as compare and integrate information during problem solving (Duncan et al., 2000). “Gifted” kids use more complex cognitive strategies to solve problems more quickly or at an earlier age than TD children (Ali et al., 2003, Athanasakis et al., 2014, Colom et al., 2004, Davidson, 1986), and the foundation for many of these advanced cognitive skills appears to be their memory system. Previous studies have shown an age‐related global increase in cortical thickness, primarily in the executive prefrontal cortex, and positive correlations have been found between the integrity of white matter connecting frontal and parietal regions in gifted children (Geake & Hansen, 2005, Gerig et al., 2001). Additionally, evidence for an enhanced or divergent neurodevelopmental trajectory has been found in mathematically gifted children, whose brains have a tendency to use right hemisphere fronto‐temporal systems in a selective and successful manner which allows them to perform exceptionally well (O'Boyle et al., 1990, O'Boyle et al., 1995, O'Boyle et al., 2005, Packard & Knowlton, 2002, Pesenti et al., 2001).

Yet, of the few existing studies, none has focused on memory systems. We are still missing critical information that may be fundamental to understanding the mechanics of their information integration systems, such as regional shape and connectomics of neural learning networks. Two parallel memory systems are of particular interest when studying neurodevelopment. Implicit learning describes the automatic learning of social, linguistic, and procedural tasks that are acquired and used without conscious effort. This mechanism allows children to create grammatically correct sentences and understand social norms, such as eye contact without explicit instruction (Gonring et al., 2017). Explicit learning involves an intentional, conscious effort to retain or access information, for example, reminiscing on old memories or reciting memorized facts. Implicit learning is essential in the early stages of neurodevelopment, whereas adults depend more on explicit memory, likely because a rule‐based approach is faster, easier to communicate, and leads to all‐or‐none mastery (Gray et al., 2003). In typical development, the subcortical structures that enable implicit learning (e.g., striatum) are developed in the first year of life, while explicit memory structures (e.g., hippocampus) take longer to mature.

Here, we investigated the neuroanatomy underlying these two parallel memory systems, explicit and implicit memory, in two samples. First, we examined subjects specifically recruited for being highly intellectually gifted (IQ >145) along with TD children. We used structural and diffusion‐weighted MRI and neuropsychological methods to compare the regional brain shape and connectivity of 12 children with average to high average IQ (90–130, mean = 124 ± 10.9; age = 10.7 ± 1.86; 58% female) and 18 with high IQ (145–170, mean = 153 ± 11.4; age = 10.2 ± 2.02; 56% female). Then, we tested the heuristic developed in this sample on a large, external database to attempt to confirm and expand upon the findings.

## 2 METHODS

### 2.1 Participants

Participants included 18 intellectually gifted children (confirmed by neurocognitive testing), all of whom displayed well‐balanced intellectual (mathematical, verbal, visuomotor, memory/concentration, and judgement/reasoning) profiles and 12 typically developing children. All procedures were in accordance with the Declaration of Helsinki, reviewed and approved by the University of California, Los Angeles (UCLA) Institutional Review Board prior to enrollment and all participants provided written informed consent. Participants were recruited from local Los Angeles schools and after‐school programs, including schools and programs for exceptionally gifted students. All participants completed written informed consent/assent. Eligibility for this study was determined at the first visit following the administration of a standardized IQ test (Stanford Binet 5 (Huang‐Pollock et al., 2011)). Exceptional giftedness was defined as IQ ≥ 145. Age, race, and gender‐matched typically developing healthy children with IQ ≥ 90 and ≤ 145 with no history of attentional, language or learning disorder were also recruited. Potential study participants were excluded from the study if any of these exclusion criteria were met: history of head injury, a seizure disorder, or other neurological or psychiatric disorders; past or current language disorder, attention deficit and hyperactivity disorder, conduct disorder, obsessive compulsive disorder, autism spectrum disorder or drug dependency; current/past placement in special education class or an IQ ≤ 84; contraindications for MRI (e.g., metal implants, pacemaker, braces or other metals affixed to the head, and pregnancy).

### 2.2 Group demographic comparison

Demographic factors (e.g., age, education) between IG and TD groups were compared using one‐way analysis of variance (ANOVA). Group differences in dichotomous factors (e.g., gender, ethnicity,) were assessed using chi‐square analyses. We used *p* < .05 as our cutoff for statistical significance for these demographic analyses.

### 2.3 Neuropsychological testing

All participants completed a standardized neuropsychological measure (Stanford‐Binet 5; Huang‐Pollock et al., 2011) designed to test IQ with a high ceiling (IQ = 170) allowing for the quantification of IQ in exceptionally intellectually gifted participants.

### 2.4 MRI acquisition

All neuroimaging data were collected using a 12‐channel head coil on a 3T Siemens Tim Trio (Siemens Medical Solution, Erlangen, Germany) MRI scanner housed at the UCLA Staglin IMHRO Center for Cognitive Neuroscience. Structural MP‐RAGE T1‐weighted scans were acquired with 120–1.0 mm sagittal slices, FOV = 256 mm (A‐P) × 192 mm (FH), matrix = 256–192, TR = 450.0 ms, TE = 10.0 ms, flip angle = 8, voxel size = 1.0 mm × 0.94 mm × 0.94 mm. DTI data were acquired using single shot spin‐echo planar imaging (EPI) and using the following parameters: TR = 8400 ms; TE = 91 ms; 1282 matrix, FOV = 256 mm, *b* = 1000 s/mm^2^, NEX = 1, 64 slices, 2 mm slice thickness, 0 mm skip, PAT = 2. Diffusion was collected in 64 directions (*b* = 1000 s/mm^2^) with 4 images with *b* = 0 s/mm^2^. All images were quality‐controlled and visually inspected prior to being preprocessed and analyzed.

### 2.5 Subcortical shape processing and analysis

3D morphometric T1‐weighted anatomical scans were collected for all participants. Using FMRIB Software Library version 6.0 (Kalbfleisch, 2004) (FSL) the T1 data were run through an automated, model‐based subcortical segmentation protocol (FSL FIRST (Kuhn et al., 2017)) using a boundary correction method. The vertex and bvar files of automatically segmented subcortical regions of interest (ROI; which included the amygdala, nucleus accumbens, caudate, hippocampus, pallidum, putamen, and thalamus) in the left and right hemisphere were then visually inspected for quality. The final vertex files for the segmented subcortical structures were concatenated both between‐group and within‐group. All automatically segmented ROIs for each participant were aligned to the same standard space. Each vertex provides data on the location of the surface of the ROI at the same point in space for each participant. In order to perform group analyses, each participant's data were registered to a standard space so that each vertex was aligned to the same point in space for group comparisons. The mean surface of the sample was used as the target for this alignment.

These scalar vertex values were then analyzed using parametric statistical analyses to investigate the relationship between‐group (IQ vs. TD) and the shape of each subcortical structure. These analyses were performed at each vertex using a Monte Carlo simulation in FSL's randomize script (Kyllonen & Christal, 1990, Mills & Tissot, 1995). Regressions assessed the relationship of radial distance at each vertex to variables of interest (i.e., group designation, IQ). A multistep, quality‐controlled processing pipeline was used to correct for multiple sources of artifact and all results were corrected for multiple comparisons (*q* > .05) using false discovery rate (FDR) (Na et al., 2007).

### 2.6 White matter microstructure processing and analysis

A routine DWI processing pipeline was carried out using FSL v 6.0 (Kalbfleisch, 2004), involving brain extraction, correction for eddy current distortion, and motion and tensor fitting. DWI metrics of white matter microstructure, fractional anisotropy (FA) and mean diffusivity (MD) were computed for each participant. Analyses were conducted using Tract Based Spatial Statistics (TBSS) (Navas‐Sánchez et al., 2014), a method allowing for voxel‐wise statistical interrogation of DWI metrics along WM tracts of interest (TOI). TOIs were derived from the John Hopkins University White Matter atlas (Neihart et al., 2002) and included bilateral: anterior thalamic radiation (ATR), inferior fronto‐occipital fasciculi (IFO), inferior longitudinal fasciculus (ILF), superior longitudinal fasciculus (SLF), uncinate fasciculus (UNC), cingulate bundle (CGC), hippocampal (HC) white matter, as well as forceps major and forceps minor. Parametric statistical analyses investigated between‐group differences in WM microstructure (FA and MD). Correlation analyses investigated the relationship between IQ and FA/MD. TBSS results were corrected for multiple comparisons with threshold free cluster enhancement—TFCE (O'Boyle, 2008)).

### 2.7 Volumetric confirmation of the explicit/implicit heuristic using an external dataset

The current sample did not include children with below average to impaired IQ scores or any atypical neurodevelopmental conditions, such as attention deficit hyperactivity disorder (ADHD) or autism. Therefore, we sought to test the explicit/implicit ration heuristic in an external, large dataset that includes this wider range of developmental trajectories. Thus, the Adolescent Brain and Cognitive Development (ABCD) project (UCLA PI: Bookheimer) was used as an external dataset to attempt to validate the explicit to implicit ration heuristic derived from the gifted adolescent analysis (described in Section 3). Age, sex, global cognitive function score from the National Institute of Health (NIH) Toolbox as well as subcortical structure volume were compiled from all available ABCD participants. The volume of the (explicit memory) brain regions found to be significantly greater in the IG group were summed to create the explicit memory brain component. The volume of the (implicit memory) brain regions found to be greater in the TD group were summed to create the implicit memory brain component. Then, the explicit/implicit ratio variable was calculated by dividing the explicit memory brain component by the implicit memory brain component. A correlation analysis was then computed assessing the relationship between this explicit/implicit memory brain ratio and global cognitive performance as measured by the NIH Toolbox. Given the format that ABCD data are made available (i.e., extracted values in spreadsheet rather than raw MRI data), we were not able to run shape analysis. However, we were able to run volume analyses. Importantly, shape analyses are more regionally specific and more sensitive than volume analysis (Rogers, 1986). As such, effects found in volume analyses will be present in shape analyses, however, shape analysis results might be present when volume analysis is not sensitive enough to detect change (Rota et al., 2009). Therefore, successful replication of the heuristic using the ABCD volumetric data should effectively replicate the shape findings from the initial sample herein.

## 3 RESULTS

### 3.1 Demographic group comparison

The IG and TD groups did not significantly differ in age, years of education, ethnicity or gender (all *p* > .05). The IG group (153 ± 11.4) had significantly higher IQ than the TD group (128 ± 10.9, *p* < .01; Table 1). The IG group was comprised of “well‐balanced” intellectual profiles. All gifted participants were at the Superior Adult 1 or higher level for math and verbal skills, with one exception. One gifted participant was at the Above Average level for “Vocabulary and Verbal Fluency” and the Superior Adult 1 for “Arithmetic Reasoning.” The same participant also tested at the Superior Adult 3 level for Judgment and Reasoning. Therefore, all IG participants appeared to be well‐balanced in mathematical and verbal proficiency.

**TABLE 1:** Demographic group comparison

	**Gifted (*N* = 18)** Mean (SD)	**Typically developing (*N* = 12)** Mean (SD)
**Age**	10.2 (2.02)	10.7 (1.86)
**Sex**	10 Female (55.6%)	7 Female (58.3%)
**IQ**	153 (11.4)	128 (10.9)

### 3.2 Subcortical shape

Significantly greater vertices were found in bilateral hippocampi and right putamen of IG whereas greater vertices were found in the left amygdala, right caudate, and bilateral nucleus accumbens in TD. Specifically, IG evidenced greater vertices in head of the left HC, the body of the right HC, and bilateral HC tail as well as posterior regions of the right putamen. TD evidenced greater vertices broadly throughout bilateral accumbens, primarily in the body of the right caudate and in the centromedian nuclei of the left amygdala. Across the entire sample, IQ was correlated with greater vertices in the bilateral HC tail (Figure 2).

### 3.3 White matter microstructure

**FIGURE 1:**
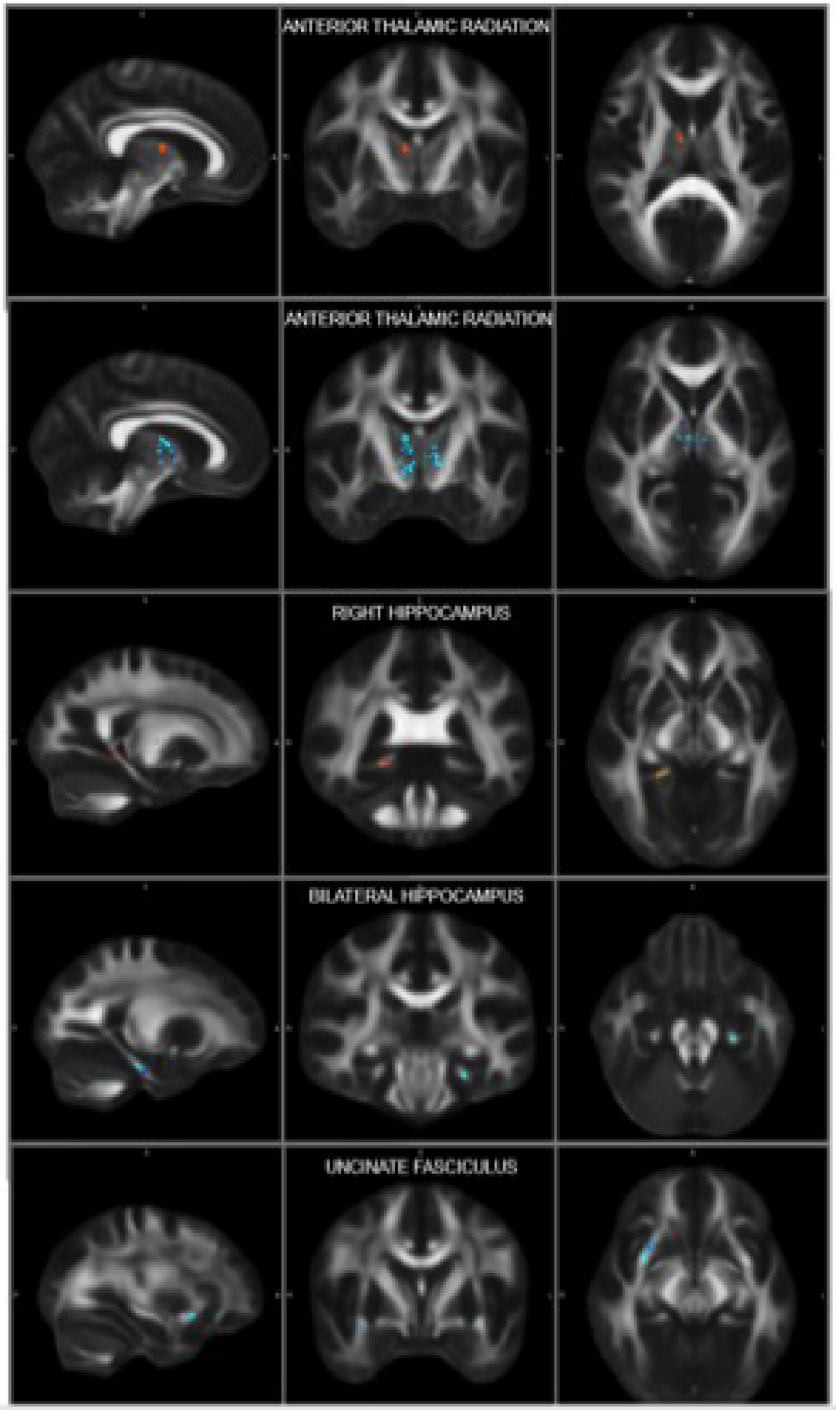
Tract based spatial statistics reveals increased white matter microstructural patency in intellectually gifted compared to typically developing children in explicit memory regions

**FIGURE 2:**
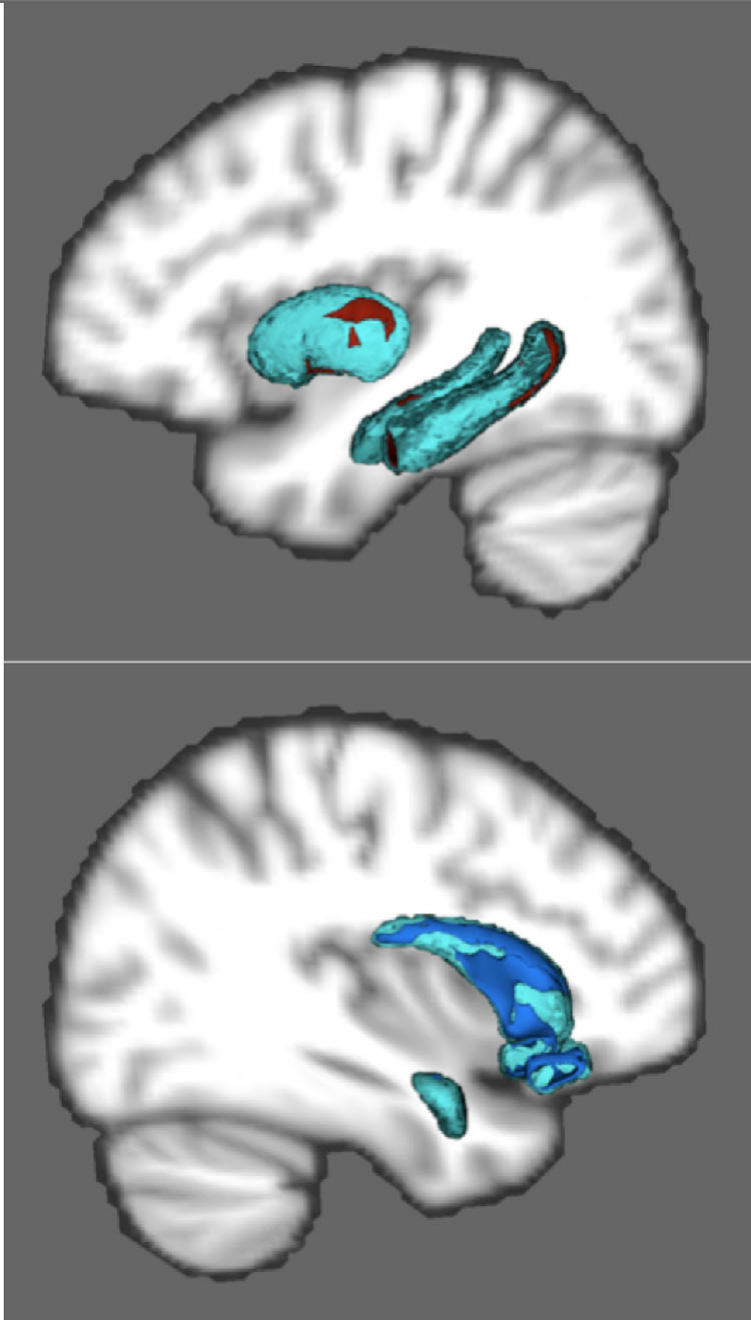
Shape analysis reveals larger shape in explicit memory regions in intellectually gifted and implicit memory regions in typically developing children

FA in the white matter of the right ATR and right HC tail (i.e., fornix) was significantly higher (.001 < *p* < .05) in the IG group compared to controls. MD was significantly lower in bilateral ATR, HC, and UNC in the IG group compared to controls. Specifically, MD results were found in a higher percentage of the right compared to left hemisphere. MD results were also found in different regions depending on hemisphere. In the HC, right hemisphere results were found near the HC tail (i.e., fornix) and left results found in the HC body (i.e., CA regions). In the UNC, findings were more inferior in the left hemisphere. Across the entire sample, FA was significantly positively correlated with IQ in bilateral (right more than left hemisphere) ATR and right HC. Further, MD was negatively correlated with IQ in bilateral (right greater than left) ATR, left HC, left UNC, left cingulate bundle, and left ILF (Figures 1 and 2).

### 3.4 Confirmation of the explicit/implicit heuristic using an external dataset

The ABCD dataset consisted of 7652 participants (mean age = 119 ± 7.5 months; 62.9% female) with all required data for this study. The explicit/implicit memory ratio was significantly positive related to global cognitive performance (*r* = 0.23, *p* < 0.05) as well as the quadratic global cognitive performance (*r* = .022, *p* < .05) (Figure 4). A stepwise hierarchical regression using linear and quadratic global cognitive performance rendered a final model [F (2, 7652) = 4.50, *p* = .011) including the quadratic global cognitive performance (*β* = 0.24, *p* = .04) as a significant predictor of the explicit/implicit ratio. Given that shape analysis is more sensitive than volume analysis, this finding using volumetrics in the ABCD data set should likewise translate to a shape analysis (which was not possible given the format of the ABCD data).

A similar regression was conducted using the global cognition measures to predict a composite variable comprised of the FA of the ATR and HC white matter. There was a significant quadratic relationship [F (2, 7562) = 13.17, *p* < .0001] between global cognition (*β* = −0.034, *p* < .001) and the composite variable comprised of FA of the right ATR and HC white matter. Finally, a stepwise regression predicted a composite variable which comprised of the MD of the UNC, HC white matter, and ATR. The quadratic global cognitive score (*β* = 0.12, *p* < .001) significantly predicted this composite MD variable [F (2, 7562) = 15.31, *p* < .0001].

## 4 DISCUSSION

This investigation found that there is a double dissociation between the size and connectivity of two separate memory systems when comparing intellectually gifted children and their TD counterparts. Relative to the TD group, the gifted children had larger subcortical structures and more highly connected white matter microstructural organization between those structures in regions associated with explicit memory and IQ: bilateral hippocampus and right putamen. Specifically, the putamen, likely via integrating connections with the prefrontal cortex, and subregions of the hippocampus associated with new learning and information integration (dentate gyrus and CA3) were larger in intellectually gifted children (Figures 1 and 2). The pronounced integrity of the white matter connections between these regions is consistent with an anatomical basis for the inherent propensity for intellectually gifted children to learn, integrate, and use explicit information rapidly and efficiently. Further, these findings may relate to the propensity for gifted children to show higher levels of intrinsic motivation to read, think, and spend time alone. Interestingly, typically developing children had more connected, larger subcortical structures in regions associated with implicit memory: striatum (i.e., caudate, nucleus accumbens) and amygdala (Figure 2; Haier et al., 1988).

**FIGURE 3:**
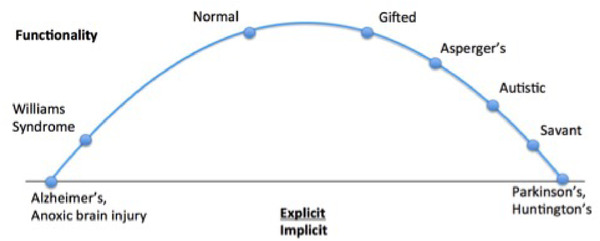
Proposed heuristic of the explicit/implicit ratio model and associated syndromes

This double dissociation suggests that intellectually gifted and typically developing children may have innately different neurodevelopmental trajectories mediated by different learning strategies. This finding led us to envision a wide spectrum of intelligence types, facilitated in part by different ratios of implicit and explicit system development. Explicit and implicit memory occupies different regions of the brain and create independent, though interconnected networks of learning. Due to limited resources and finite real estate in the brain, there may be a trade‐off between developing explicit and implicit memory systems. The differences we found may reflect a large distribution of memory system variation, with TD and gifted kids representing the most functional niches (Figure 3).

**FIGURE 4:**
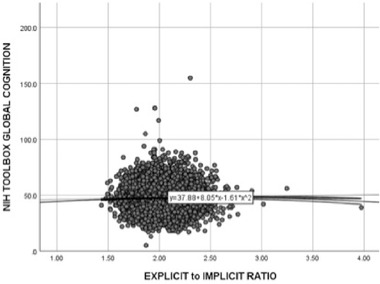
Validation of explicit/implicit ratio in large, external dataset

Findings from a mathematical prodigy's brain were previously postulated to be driven by the highly efficient episodic memory encoding and retrieval (Scharnowski et al., 2015). For this to be true, episodic memory structures as well as frontal lobe systems involved in attention and retrieval would be required. The present study reports just that: large and robustly connected episodic memory structures and frontal white matter tracts (e.g., ATR). The ATR finding is also consistent with a host of previous work which has found that high intelligence is associated with engagement of prefrontal and cingulate brain networks (Kuhn et al., 2017, Schmithorst et al., 2005), as well as previous work which found that mathematically gifted brains had higher FA in the corpus callosum and association tracts which connect the frontal lobe to basal ganglia structures, including the ATR, and fronto‐temporal/parietal tracts, including the UNC (Shaw et al., 2006). Enhanced bilateral brain regions found herein are in line with previous findings indicating that unique, bilateral brain regions are activated by mathematically precocious children during mental rotation tasks (Squire et al., 1993, Weiskopf, 2012). Additionally, the tendency for findings to be lateralized is in line with prior findings that suggest that hemispheric laterality, particularly the right hemisphere, as well as enhanced coordination within and between brain regions, is an important neural underpinning of intellectual giftedness (O'Boyle et al., 1995). Finally, the results across the entire sample suggesting IQ was correlated with DTI metrics in the ATR, HC, and UNC are in line with previous findings in a study of mathematical giftedness (Shaw et al., 2006), which reported corpus callosum, fornix, and association tract DTI metric correlations with intelligence. This white matter–IQ relationship is potentially driven, at least in part, by the gene family *plexin*, which was recently found in a genome wide association study to predict IQ (Winberg et al., 1998). Plexins are known to be linked to guidance of developing axons (Worzfeld & Offermanns, 2014), neural connectivity (Zabaneh et al., 2018), and regeneration (Zhang et al., 2017), and thus may be related to the white matter findings in our IG group.

This first sample did not include children with below average to impaired IQ scores or any atypical neurodevelopmental conditions, such as ADHD or autism. These groups may lie at the further ends of the spectrum, where explicit or implicit memory may be under‐developed or over‐developed to a detrimental extent. Thus, we then replicated this analysis in a much larger sample which did include children with below average to impaired IQ scores and some developmental differences (e.g., ADHD). We tested this explicit/implicit heuristic using a large, external data set: the ABCD project (*N* = 7652). In line with our hypothesis, we found a significant quadratic relationship between the explicit/implicit heuristic and IQ. This replication of our initial findings suggests that there may in fact be a developmental balance of implicit and explicit memory systems that can phenotypically express with different cognitive profiles. Future research may want to investigate the relative development of implicit and explicit structures in other populations, as has been thoroughly explored in neurodegenerative disorders such as Alzheimer's disease (degenerating explicit memory anatomy and skills with preserved implicit memory) and Parkinson's disease (degenerating implicit memory anatomy and skills with preserved explicit memory).

Identifying the structural and functional markers of giftedness will help us understand the deeper systems that allow children to learn inside and outside the school setting. Both implicit and explicit learning are essential, and deficits in either may lead to social, academic, and professional difficulties. Although “gifted” children score well on IQ tests, many also suffer from learning disabilities that hinder them in non‐explicit learning‐based tasks, and most TD kids could benefit from further explicit learning development (Hayden et al., 2020). This study provides some insight into how future interventions can target explicit or implicit systems to maximize learning across the developmental spectrum. We may find that brain imaging will eventually allow us to design educational interventions based on an individual's brain structure, to support healthy intellectual and behavioral development. Empirically‐grounded cognitive training procedures, such as the Program for Education and Enrishment of Relational Skills (PEERS; Laugeson et al., 2012 ), could help in boosting TD kids, gifted kids, and at‐risk clinical populations (e.g., autism spectrum disorder, learning disorder, attention‐deficit hyperactivity disorder and intellectual disability), thus allowing educational institutions to support children of all learning types and abilities. We hope this study inspires further investigation into the structures that facilitate implicit and explicit learning, their role in developmental differences, and possible future learning therapies.

## CONFLICT OF INTEREST

The authors report no conflicts of interest.

### PEER REVIEW

The peer review history for this article is available at https://publons.com/publon/10.1002/brb3.2348

## Data Availability

The data that support the findings of this study are available from the corresponding author upon reasonable request.
